# The efficient synthesis and purification of 2′3’- cGAMP from *Escherichia coli*

**DOI:** 10.3389/fmicb.2024.1345617

**Published:** 2024-03-08

**Authors:** Rohan Kulkarni, Vijay Maranholkar, Nam Nguyen, Patrick C. Cirino, Richard C. Willson, Navin Varadarajan

**Affiliations:** ^1^Department of Chemical and Biomolecular Engineering, University of Houston, Houston, TX, United States; ^2^Department of Biology and Biochemistry, University of Houston, Houston, TX, United States

**Keywords:** STING agonists, 2′3’-cGAMP production, *Escherichia coli* bacterial expression, lipopolysaccharide removal, murine cGAS enzyme

## Abstract

Agonists of the stimulator of interferon genes (STING) pathway are being explored as potential immunotherapeutics for the treatment of cancer and as vaccine adjuvants for infectious diseases. Although chemical synthesis of 2′3’ - cyclic Guanosine Monophosphate–Adenosine Monophosphate (cGAMP) is commercially feasible, the process results in low yields and utilizes organic solvents. To pursue an efficient and environmentally friendly process for the production of cGAMP, we focused on the recombinant production of cGAMP via a whole-cell biocatalysis platform utilizing the murine cyclic Guanosine monophosphate–Adenosine monophosphate synthase (mcGAS). In *E. coli* BL21(DE3) cells, recombinant expression of mcGAS, a DNA-dependent enzyme, led to the secretion of cGAMP to the supernatants. By evaluating the: (1) media composition, (2) supplementation of divalent cations, (3) temperature of protein expression, and (4) amino acid substitutions pertaining to DNA binding; we showed that the maximum yield of cGAMP in the supernatants was improved by 30% from 146 mg/L to 186 ± 7 mg/mL under optimized conditions. To simplify the downstream processing, we developed and validated a single-step purification process for cGAMP using anion exchange chromatography. The method does not require protein affinity chromatography and it achieved a yield of 60 ± 2 mg/L cGAMP, with <20 EU/mL (<0.3 EU/μg) of endotoxin. Unlike chemical synthesis, our method provides a route for the recombinant production of cGAMP without the need for organic solvents and supports the goal of moving toward shorter, more sustainable, and more environmentally friendly processes.

## 1 Introduction

**Figure 1:**
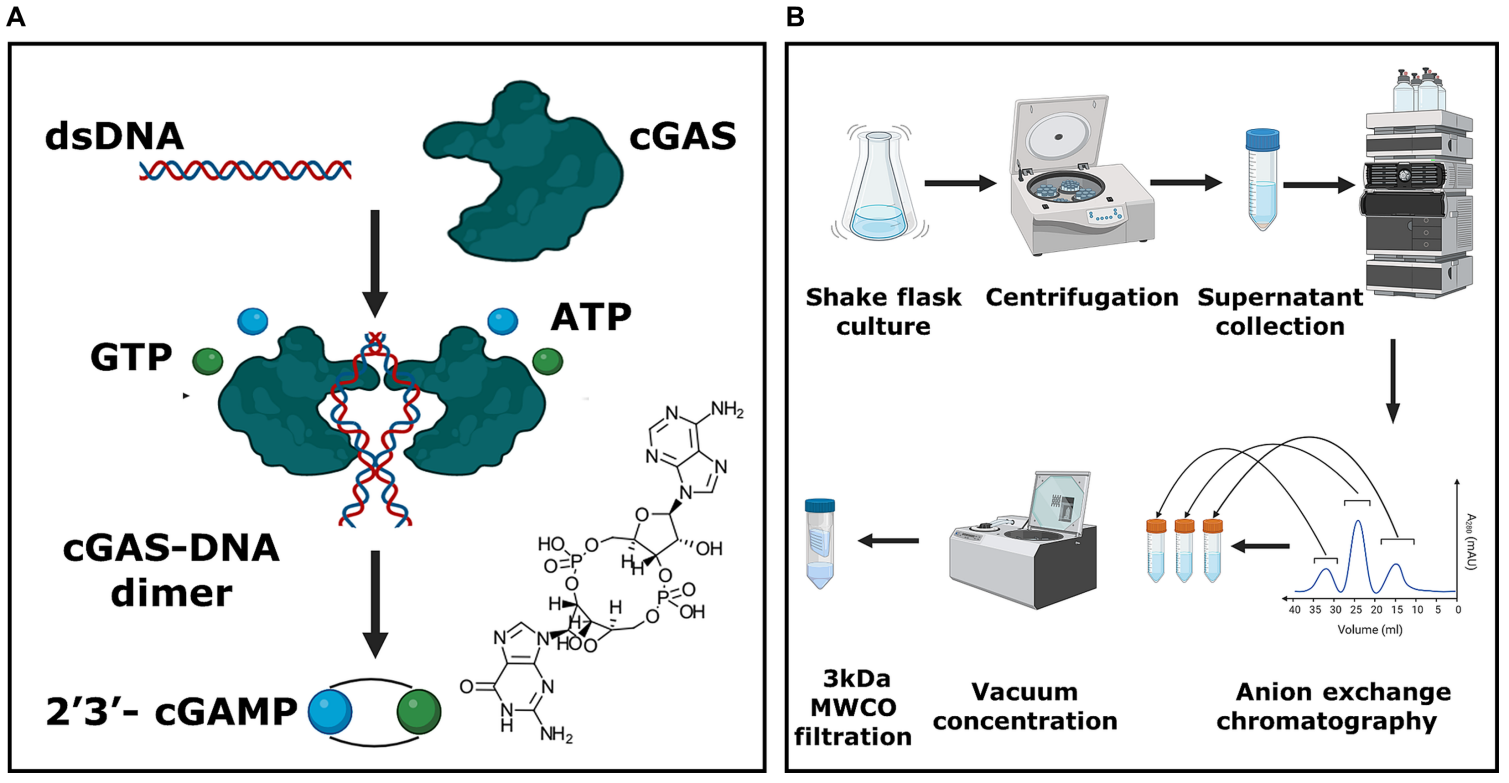
*E. coli*-based whole-cell production platform for cGAMP. **(A)** Schematic representing the enzymatic reaction responsible for the synthesis of 2′3’-cGAMP. **(B)** The overall process for the production and purification of 2′3’-cGAMP. The unit process begins with the microbial culture of *E. coli* BL21 (DE3) cells, followed by harvesting the supernatant. This supernatant is purified utilizing anion exchange chromatography, and the eluate fractions are concentrated using a vacuum centrifuge. Subsequently, the concentrated eluate is filtered using a 3 kDa MWCO filtration system to yield the final cGAMP product.

Double-stranded DNA (dsDNA) in the cytoplasm of mammalian cells acts as a potent danger signal indicative of either pathogenic infection due to viruses or bacteria, cellular damage, or cancer (Hopfner and Hornung, 2020; Chen and Xu, 2022). This mislocalized dsDNA allosterically activates cyclic Guanosine Monophosphate Adenosine Monophosphate (GMP-AMP) synthase (cGAS) which synthesizes the cyclic dinucleotide, 2′3’- cyclic Guanosine Monophosphate–Adenosine Monophosphate (hereafter cGAMP) (Hopfner and Hornung, 2020; Chen and Xu, 2022; Ritchie et al., 2022; Figure 1A). cGAMP binds to the stimulator of interferon genes (STING), a membrane protein, leading to the oligomerization of STING and its translocation to the trans-Golgi network, initiating a signaling cascade that culminates in the secretion of type I and type III interferons (Barber, 2015; Ritchie et al., 2022). cGAMP acts as the messenger that ties the recognition of dsDNA by cGAS to the subsequent activation of STING (Barber, 2015; Ritchie et al., 2022). cGAMP is easily exported out of the cell and hence functions as a soluble extracellular immunotransmitter (Ritchie et al., 2019; Carozza et al., 2020). This ensures that the effect of recognition of mislocalized dsDNA is transmitted to all cells within the microenvironment, facilitating a broad innate immune response. As a result, cGAMP and STING agonists are being actively investigated as immunotherapeutics for the treatment of cancers and as mucosal adjuvants for the development of vaccines against infectious diseases (Gogoi et al., 2020; An et al., 2021; Petrovic et al., 2021; Wu et al., 2022).

The chemical synthesis of cGAMP has been accomplished through several pathways (An et al., 2021; Bartsch et al., 2022). The phosphoramidite-based synthesis is an integrated eight-step one-flask synthesis and is the most commonly used route but suffers from multiple drawbacks including low yields (5%) and the need to use hazardous organic solvents (Gaffney et al., 2010). As an alternative to chemical synthesis, heterologous cGAS, expressed and purified from *E. coli* cells, is used for the enzymatic biosynthesis of cGAMP (Becker et al., 2021a,b). The cGAS enzyme is incubated with dsDNA as an allosteric activator and converts Adenosine Triphosphate (ATP) and Guanosine Triphosphate (GTP) into cGAMP with 85–90% yield in HEPES buffer (Becker et al., 2021a). The advantage of enzymatic synthesis is that it does not need organic solvents and results in high-yield conversion (Rolf et al., 2019; Rosenthal et al., 2020). A process Life Cycle Assessment (LCA) has also shown enzymatic synthesis to be the more environmentally friendly process (Becker et al., 2023). The disadvantage of enzymatic biosynthesis however is that both the substrate GTP and the expression and purification of cGAS are expensive.

To overcome the need to purify cGAS, intact *E. coli* cells transformed with murine cGAS (mcGAS) have been used as biocatalysts for the production of cGAMP. In this report, they demonstrated that surprisingly, cGAMP was secreted into the culture medium (Lv et al., 2019). This overall workflow is attractive since the ATP and GTP substrates are available intracellularly, and the desired cGAMP product accumulates in the supernatant. There are, however, drawbacks to this process. First, the downstream process utilizes a protein affinity chromatography step (STING-ligand binding domain protein, STING-LBD). As stated above, this in turn necessitates the expression and purification of STING-LBD which increases process economics and environmental impact. Purification steps that do not rely on protein-affinity chromatography are desirable. Second, the process workflow does not remove endotoxin. One of the primary disadvantages of using *E. coli* cells as biocatalysts is the need to eliminate endotoxin since it will interfere with all downstream immunological applications. It is thus highly desirable to remove the endotoxin from the final cGAMP product.

In this study, we systematically tested the impact of (1) culture conditions on the yield of cGAMP secreted from *E. coli* BL21(DE3) cells, (2) amino acid substitutions in mcGAS shown to reduce DNA dependence for activation, and (3) the comparative ability of plasmid and *E. coli* genomic DNA to activate mcGAS. We developed a single-step purification protocol that relies only on anion exchange chromatography and results in 60 ± 2 mg/L of purified cGAMP with <20 EU/mL of endotoxin. The high yield of endotoxin-free cGAMP with the aid of simple purification makes the process attractive for fermentation-based production of cGAMP on a large scale.

## 2 Materials and methods

### 2.1 Plasmid design

The nucleic sequence encoding full-length mcGAS was downloaded from GenBank (Accession Code: NP_775562). We performed codon optimization and the mcGAS gene block was synthesized at IDT (Coralville, Iowa). We cut pET28A (+) plasmid at NdeI and XhoI cut sites and inserted the gene block sequence consisting of an N-terminal SUMO tag and mcGAS full-length gene along with overhangs into the plasmid backbone using NEBuilder® HiFi DNA assembly master mix (E2621L, New England Biolabs). Bacterial transformation and plasmid propagation was done via electroporation in electrocompetent E. cloni EC10 cells (Agilent Inc.).

### 2.2 *Escherichia coli* strains and bacterial cell culture

The study included testing multiple bacterial strains of *E. coli* to examine productivity including E. cloni EC10; MG1655 (DE3); and *E. coli* Bl21 (DE3) and BL21(DE3) RIL (Agilent Inc.). These strains were grown similarly to a procedure described previously (Lv et al., 2019). Briefly, agar plates containing the appropriate resistance marker (50 μg/mL Kanamycin sulfate) were streaked for single colonies, which were then picked and seeded into 5 mL modified M9 medium (minimal M9 salts supplemented with 0.8% glucose, 5 mM MgSO_4_, 0.1 mM CaCl_2_, and 0.01 mM ferrous sulfate) overnight at 37°C. The overnight culture was seeded for a target OD_600_ of 0.05 in a 100 mL modified M9 medium. This culture was shaken at 250 rpm at 37°C and was induced after 6–8 h at OD_600_ value of 0.6 to 0.8 with 0.1 mM IPTG. Cultures were harvested once OD_600_ reached a value of 4 at 16–20 h. The culture was spun at 4000 × g for 45 min at 4°C, the supernatant was filtered using a 0.2 μm filter (VWR LLC, PA), and the cell pellet and supernatant were stored separately at −80°C.

### 2.3 High-performance liquid chromatography

To quantify concentrations of 2′3’- cGAMP produced in bacteria culture, we generated an HPLC standard curve for various concentrations of 2′3’- cGAMP fitted to a standard linear regression. The chromatography and peak analyses were done on a Shimadzu HPLC system and SPD-20A UV detector using LCsolution software (version 1.25) respectively. The commercial standard of 2′3’-cGAMP was obtained from ChemieTek (Indianapolis, US) and used without further purification. A 20 mg/mL stock solution of 2′3’-cGAMP was prepared by dissolving the 2′3’-cGAMP powder in distilled water, and further diluted to produce standard concentrations (7.81, 15.62, 31.25, 62.5, 125, 500, and 1,000 μM). Each sample was injected with 20 μL volume per HPLC run (UV 256 nm). For the HPLC method, the solvents consisted of Acetonitrile (A), and 5 mM Ammonium Acetate (B). The flow rate was maintained at 1 mL/min and the initial composition was 1.5% A and 98.5% B. The mobile phase composition was linearly increased up to 10% A at 7.5 min and to 30% A at 10 min. Flow composition returned to 1.5% A at 12.5 min. A 2′3’-cGAMP HPLC standard curve was prepared by plotting the 2′3’-cGAMP concentration vs. the corresponding peak areas (mAU x min) (Table 1).

**Table 1:** Time plot for solvents in cGAMP quantification HPLC method.

Time (min)	% A (Acetonitrile)	% B (Ammonium acetate)
0	1.5	98.5
7.5	10	90
10	30	70
12.5	1.5	98.5

### 2.4 Liquid chromatography–mass spectrometry (LC-MS)

To confirm the presence of 2′3’- cGAMP and validate our findings for 2′3’- cGAMP productivity, we performed LC–MS using a previously published method (Carozza et al., 2020). 2′3’-cGAMP from Chemie Tek (Indianapolis, US) was used as a standard at 10–100 μg/mL. Samples were analyzed using an AB Sciex QTRAP® 4,000 LC–MS/MS system. A volume of 10 μL was injected into a Biobasic® AX LC column (5 μm, 50 × 3 mm; ThermoFisher Scientific, MA). The solvents consisted of 100 mM ammonium carbonate (A) and 0.1% (v/v) formic acid in acetonitrile (B). The initial condition was 90% B, maintained for 0.5 min. The mobile phase was ramped linearly to 30% A from 0.5 min to 2.0 min, maintained at 30% A from 2.0 min to 3.5 min, ramped linearly to 90% B from 3.5 min to 3.6 min, and maintained at 90% B from 3.6 min to 5 min. The flow rate for samples was set to 0.6 mL/min. The mass spectrometer was operated in electrospray positive-ion (ESI) mode with the source temperature set at 500°C. Declustering and collision-induced dissociation were achieved with nitrogen gas at a medium flow rate. Declustering and collision-induced dissociation were achieved with nitrogen gas. For each molecule, the Multiple Reaction Monitoring (MRM) transition (s) (*m*/*z*) are listed in Table 2.

**Table 2:** MRM transition(s) (m/z) parameters for 2′3’- cGAMP.

MRM pair MW (Da)	DP	Collison Energy 1 (eV)	Collision Energy 2 (eV)	Collision Energy 3 (eV)	C_exp_	Optimized XIC (*10^5^) (eV)
136	190	43	44	48	9	3
312		54	55	55	13	9
330		43	42	40	6	4
476		38	39	38	12	6
523		30	29	29	30	11
152		41	39	40	10	3.9

### 2.5 Anion exchange chromatography

Our anion exchange chromatography method consisted of Buffer A (20 mM Tris, pH 7.5) used for loading and washing; and Buffer B (20 mM Tris, NaCl (50–125 mM), pH 7.5). A HighQ® resin (Bio-Rad, CA) column (1 mL) was equilibrated with 25 column volumes (CV) of buffer A on an Äkta® Explorer system (Pharmacia) and monitored using a UV detector (256 nm). The bacterial supernatant post-centrifugation and filtration was mixed with buffer A in a 1:5 volume ratio to reduce the conductivity of the load to 5 mS/cm. The diluted supernatant (25 CV) was then loaded into the column. The column was washed with buffer A (25 CV) to remove impurities. Increasing concentrations of NaCl in buffer B (50 mM NaCl, 12.5 CV; 100 mM NaCl, 10 CV) were applied to selectively elute 2′3’-cGAMP. All chromatography steps were conducted at a flow rate of 0.5 mL/min.

### 2.6 Lipopolysaccharide measurement and removal

We measured the Lipopolysaccharide (LPS) concentrations at various stages of the production and purification of 2′3’-cGAMP using the Pierce® Endotoxin kit (ThermoFisher, NJ). Amicon® Ultra 3 kDa Molecular Weight Cut Off (MWCO) filters (MilliPore Sigma, MA) were used according to manufacturers’ instructions to reduce Lipopolysaccharide (LPS) concentration from chromatography eluate pools that had been vacuum concentrated previously. Using the Pierce® Endotoxin kit (ThermoFisher, NJ), a standard curve was prepared using standards for 1.0, 0.5, 0.25, and 0.1 EU/mL; after which dilutions of actual experimental samples (10x, 100x, 1,000x) were prepared with endotoxin-free water provided with the kit. Absorbance readings were taken at 405 nm and the readings which lie in the value range for the standard curve were noted. The actual concentrations of LPS in the samples were calculated using a linear regression equation generated from the standard curve.

### 2.7 Cell lysate preparation and Western blotting

For cell disruption, the pellets were prepared using a previously published method (Rolf et al., 2019). Cell pellets after supernatant separation were thawed on ice and resuspended in 10 mL lysis buffer (50 mM Tris–HCl (pH 8), 300 mM NaCl, 40 mM imidazole, 1 mM TCEP). Five cycles of sonication (Branson 2,800™) were performed for 30 s each, and the sample was kept on ice throughout the process. Cellular debris was removed by performing centrifugation twice at 43,000 × g for 15 min at 4°C. Soluble components were sterile filtered (0.2 μm) and protein concentration in cell lysates was measured using a standard BCA assay.

An SDS-PAGE was performed on the cell lysates using a 4–15% gradient Bio-Rad™ gel (Cat:456–1,084); cell lysates were loaded 25 μg each into all the wells, and the gel was run under reducing conditions at 100 V for 2 h at 4°C in a running buffer (Tris base 25 mM, glycine 192 mM, SDS 0.1%). Gel transfer was done at 90 V for 1 h at 4°C onto a polyvinylidene fluoride (PVDF) membrane in transfer buffer (0.0375% SDS, 200 mL methanol, 800 mL DI water). Blocking was done for 2 h using a blocking solution (1x Tris-buffer Saline 0.1% Tween-20 [TBST], 5% non-fat milk). The membrane was washed 3x with 1x TBST (100 mL 10x TBS, 1 mL Tween 20® detergent (Sigma Aldrich™, Cat: P1379), 900 mL DI water), and then incubated with Murine anti-SUMO tag Ab (Genscript™) 0.005 μg/mL dissolved in 0.45 μm filtered 1x TBST with 2.5% BSA (FisherScientific™ Cat: BP1600-100) overnight as the primary antibody incubation, followed by 3x wash with 1x TBST and 1 h secondary antibody incubation with anti-mouse IgG HRP conjugated antibody. The blot was finally developed by soaking it in Pierce™ 1-Step™ TMB substrate solution (Thermo Scientific™, Cat: PI37574) for 10 min to develop the PVDF membrane.

### 2.8 mcGAS activation and enzymatic activity measurement

To study cGAS activity and to compare the amounts of genomic and plasmid DNA required to activate mcGAS, we designed an experiment around fixed mcGAS, ATP, and GTP amounts while diluting DNA required for enzymatic activation. Murine cGAS enzyme (BellBrook labs™, Cat: 2239), ATP (Fisher Scientific™, Cat: R0441), and GTP (Fisher Scientific™, Cat: R0461) were used at 0.1 μM, 100 μM, and 100 μM respectively, in a 40 μL reaction volume. The genomic DNA was isolated, and gel extracted from *E. coli* BL21 (DE3) culture and the pET28A(+) plasmid with the immunostimulatory DNA sequence proven to activate mcGAS (Zhou et al., 2018) was prepared using Qiagen Miniprep™ (Cat: 27104), respectively. The enzymatic reactions were conducted at 37°C for 1 h in 20 mM Tris–HCl, pH 7.5, 150 mM NaCl, 5 mM MgCl_2_, 1 μM ZnCl_2_, and 0.01% Tween-20. The final 2′3’-cGAMP concentration in the reaction mixture was measured using HPLC. The efficiency of various ATP and GTP concentrations to activate mcGAS was quantified *in vitro.* The enzymatic reaction was carried out at varying concentrations of ATP (100, 200, 300, 400 μM), GTP (100, 200, 300, 400 μM), with fixed concentrations of mcGAS (0.1 μM) and plasmid dsDNA (2 nM). The cGAMP produced was measured at the end of the reaction using the standard HPLC assay (256 nm).

### 2.9 THP-1 dual cell assays

We cultured Tohoku Hospital Pediatrics-1 (THP-1) dual™ cells (NF-κB-SEAP IRF-Luc Reporter Monocytes) [InvivoGen, San Diego, CA, Cat: thpd-nfis] in a humidified incubator at 37°C with 5% CO_2_. We grew them in RPMI 1640 medium, supplemented with 2 mM L-glutamine, 25 mM HEPES, 10% heat-inactivated fetal bovine serum (heated for 30 min at 56°C), 100 μg/mL Normocin™, and Pen-Strep (100 U/mL-100 μg/mL). To maintain a positive selection of reporters, we grew the THP-1 Dual cells in the presence of 100 mg/mL zeocin (InvivoGen®, Cat: ant-zn-1) and 10 mg/mL blasticidin (InvivoGen®, Cat: ant-bl-1).

We carried out THP-1 cell stimulation experiments following manufacturer guidelines (InvivoGen®, CA, United States). First, we placed 0.1 million cells into each well of a 96-well plate, adding 180 μL of growth media. Next, we treated the cells with our samples (20 μL each) and incubated them at 37°C for 12 h. To quantify luciferase activity, we harvested 10 μL of culture fluid from each well at 12 h and added 50 μL of QUANTI-Luc™ (InvivoGen®) substrate solution to every well in a dark environment. The luminescence was measured using Cytation 7 (Bio-Tek Instruments, Inc.).

### 2.10 Flow cytometry assays

Propidium iodide (PI) staining kits were purchased from Thermo Fisher (Waltham, MA). 0.2 μm filtered 0.85% NaCl (Fisher Scientific, Atlanta, GA) solution was used for preparing bacterial cell culture samples. Bacterial cell cultures from different time points were centrifuged at 4500 × g for 30 min, resuspended in 0.85% NaCl, and diluted 100-fold to obtain samples for PI staining. PI was added to the samples with a final concentration of 20 μM. The samples were then incubated for 15 min at 37°C in the dark before being analyzed with a flow cytometer (NovoCyte™ Flow Cytometer, NovoCyte™ 3000RYB, ACEA Biosciences Inc., San Diego, CA). Forward and side scatter parameters of unstained controls were used to gate the cell populations on flow diagrams. Cells whose membranes were compromised by treating with 70% ethanol for 1 h served as a positive control. Cells were excited at a wavelength of 561 nm for red fluorescence and a 615/20 nm bandpass filter was used.

## 3 Results

### 3.1 Optimizing the yield of cGAMP in *Escherichia coli* culture supernatants

Our objective was to design a method for the recombinant production and purification of 2′3’-cGAMP from intact *E. coli* cells expressing the cGAS enzyme (Figures 1A,B). The comparisons of cGAS and their variants from different species have shown that the murine cGAS (mcGAS) has the highest specific activity among multiple species homologs, with >90% conversion of ATP to cGAMP (Rolf et al., 2019). For this reason, we decided to clone mcGAS and added an N-terminal SUMO tag that is known to specifically promote the stability of mcGAS and enable soluble expression in *E. coli* (Hu et al., 2016; Lv et al., 2019). Due to the high frequencies of rare codons encoding Arg, Ile, and Leu in mcGAS; others have expressed mcGAS in the *E. coli* BL21(DE3)-RIL strain (Lv et al., 2019). This strain contains additional copies of argU, ileY, and leuW tRNA genes to facilitate the expression of heterologous proteins containing high frequencies of the rare codons that encode for Arg, Ile, and Leu. To eliminate the need for a specialized strain of *E. coli,* we synthesized a codon-optimized mcGAS gene to eliminate the rare Arg (AGG), Ile (AUA), and Leu (CUA) codons. We cloned the gene into the T7 plasmid, pET28a, and transformed the plasmid into three *E. coli* strains: BL21(DE3)-RIL, BL21(DE3), and MG1655 (DE3) [K12 strain].

**Figure 2:**
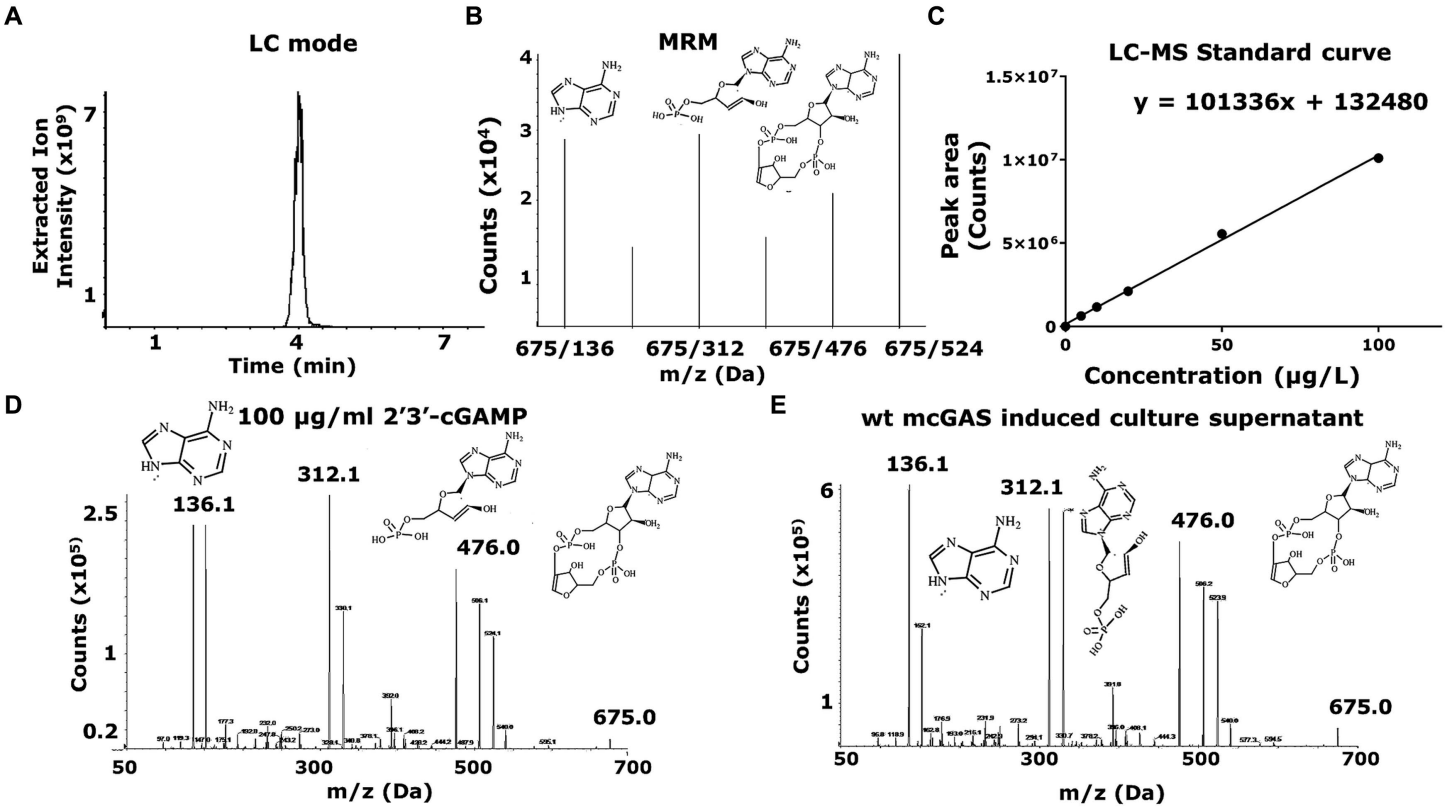
LC–MS-based identification and quantification of 2′3’-cGAMP shows cGAMP being secreted to the bacterial supernatant. **(A)** The LC–MS was set up to detect a 2′3’-cGAMP peak in LC mode. The HPLC chromatogram for samples including cGAMP standard (10–100 μg/mL) and mcGAS culture supernatants was plotted using the extracted ion intensity of characteristic daughter molecules against time. **(B)** The mass spectrometer (AB Sciex QTRAP® 4,000 LC–MS/MS) was set up in a multiple reaction monitoring (MRM) mode for the peak, where the chemical species would be broken down into characteristic daughter molecules, indicated by the multiple peaks at 136 Da, 312 Da, 476 Da and 524 Da, among others. The plots measure ion counts (x10^5^) for each individual daughter molecule. **(C)** A standard curve of 2′3’-cGAMP concentration was determined with cGAMP concentrations (10–100 μg/mL) against the area under the curve in the LC mode peak, with the peak areas corresponding to the extracted ion intensity of the characteristic daughter molecule (476 Da) of cGAMP. **(D,E)** A comparison between the MRM in LC–MS mode for the bacterial supernatant versus standard 2′3’-cGAMP solution showed that *E. coli* expression derived 2′3’-cGAMP (675.1- > 476.1) was identical to the chemically synthesized cGAMP, confirming the identity of the molecule.

**Figure 3:**
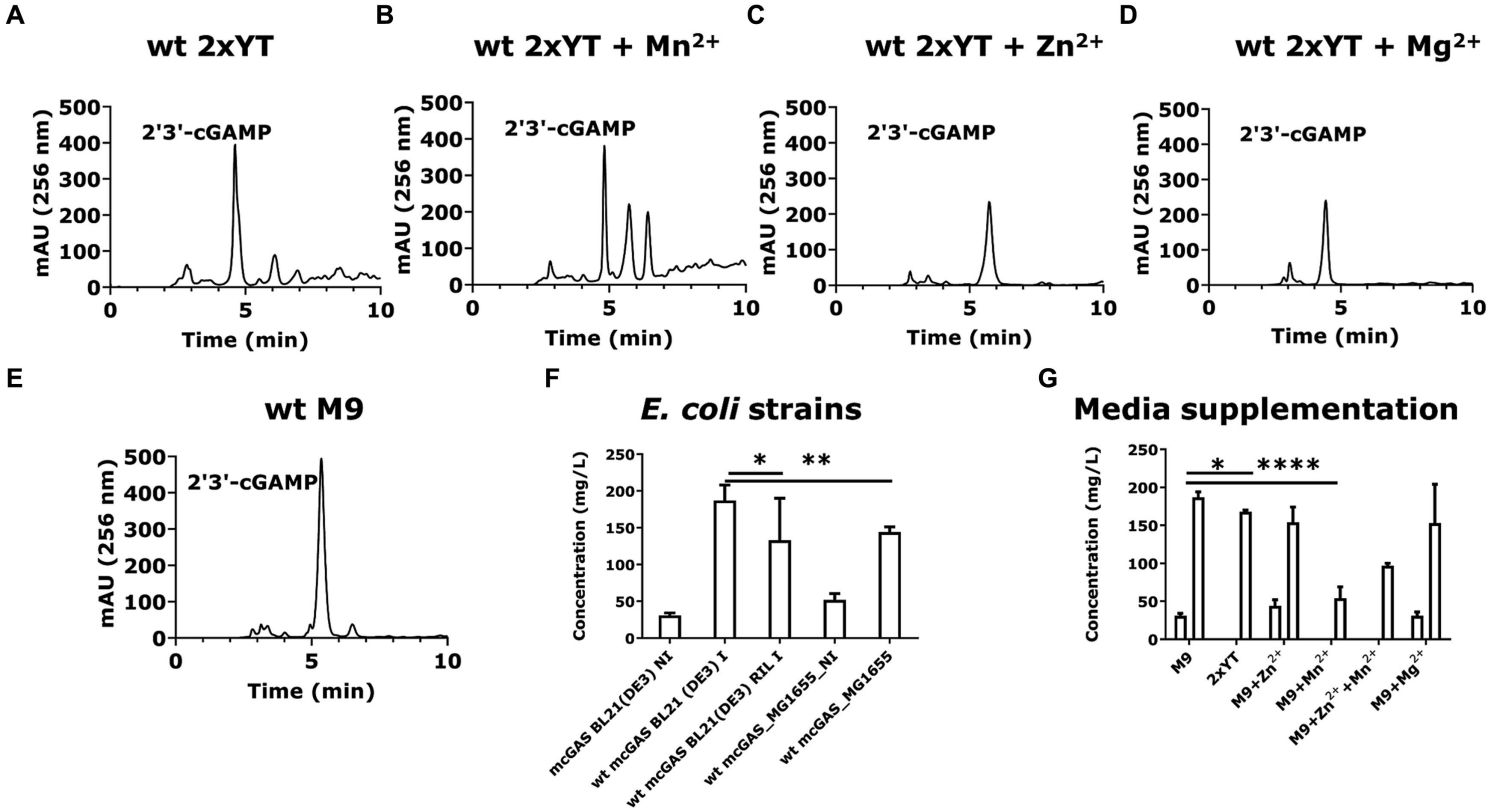
*E. coli* BL21(DE3) cells expressing wt-mcGAS in modified M9 secrete high amounts of 2′3’-cGAMP. **(A–E)** Representative HPLC chromatograms (256 nm) assaying cGAMP in the culture supernatants derived from *E. coli* BL21(DE3) cells expressing wt-mcGAS plasmid grown in 2 × YT, 2 × YT supplemented with Mn^2+^, 2 × YT supplemented with Zn^2+^, 2 × YT supplemented with Mg^2+^, or modified M9 medium. **(F,G)** The bar plots show the comparison of the HPLC-measured cGAMP concentrations in bacterial culture supernatants among multiple bacterial strains and media supplementations. The area under the curve (mAU × min) from chromatograms of the supernatant samples was plotted against those of cGAMP standards to obtain the cGAMP concentration in the supernatants. * Bar plots show the average measurement derived from at least three independent cultures (*N* = 3) and the error bars show the SEM. Statistical significance indicated by (*) was performed for dataset comparisons using unpaired, two-tailed t-test and Welch’s correction. (*) indicates a value of *p* < 0.05.

As expected, the growth of the *E. coli* BL21(DE3) cells in M9 minimal medium led to the secretion of cGAMP into the supernatants. To enable the accurate detection of cGAMP and distinguish it from 3′3’-cGAMP, c-di-AMP, and c-di-GMP (alternate products), we used a previously optimized method based on LC–MS/MS (Carozza et al., 2020). We used chemically synthesized 2′3’ cGAMP as the standard for LC–MS/MS. Although both cGAMP and 3′3’-cGAMP display identical mono-protonated ions at m/z of 675.1, cGAMP produces a unique product ion of m/z 476.1 upon fragmentation, and this product ion can be used to fingerprint cGAMP (Figures 2A–E). The mass scan for the recombinant expression-derived cGAMP (675.1- > 476.1) was identical to the chemically synthesized cGAMP and this confirmed the identity of the molecule. To enable routine monitoring of cGAMP in culture supernatants, we used a reverse phase HPLC method and this again demonstrated that cGAMP was secreted in the supernatants of the bacterial culture (Figures 3A–E).

We confirmed that the cGAMP in the supernatant was not released solely due to cell death by examining the viability of the induced and non-induced cell cultures. Under both conditions, cell viability was >80% throughout the induction period (Supplementary Figures 1A,B). To confirm that the secretion of cGAMP was the dominant source of cGAMP, we also evaluated the total cGAMP in both the supernatants and the bacterial pellets. Quantification using HPLC confirmed that the cGAMP within the bacterial pellets (12 ± 5 mg/L) was much lower than the cGAMP in culture supernatants (186 ± 7 mg/mL; Supplementary Figure 1C).

We tested the different strains for the production of cGAMP. *E. coli* BL21(DE3) cells yielded 186 ± 7 mg/L of cGAMP in the supernatant, which was ~1.5-fold greater than *E. coli* BL21(DE3)-RIL cells (130 ± 30 mg/L; Figure 3F). *E. coli* K12 strain MG1655 (DE3) produced an intermediate amount of cGAMP, 140 ± 5 mg/L (Figure 3F). These results established that with the codon-optimized version of SUMO-mcGAS, we can achieve efficient production of cGAMP in *E. coli* BL21(DE3) cells. We next sought to determine the impact of media composition on the yield of cGAMP secreted from SUMO-mcGAS-*E. coli* BL21(DE3) cells. Comparisons of the complex medium (2 × YT) and M9 minimal medium showed that the yield of the cGAMP in supernatant was lower with 2 × YT 170 ± 2 mg/L (Figure 3G). We thus prioritized SUMO-mcGAS- *E. coli* BL21(DE3) cells cultured in M9 for all further experiments.

Divalent cations Mn^2+^, Zn^2+^, and Mg^2+^ are known activators of mcGAS, which act using distinct mechanisms (Du and Chen, 2018; Zhao Z. et al., 2020). The addition of Mn^2+^ has been shown to increase the sensitivity of the cGAS-STING pathway through allosteric DNA-binding (Wang et al., 2018; Hooy et al., 2020), while Mg^2+^ and Zn^2+^ both are natural cofactors of the mcGAS enzyme (Gao et al., 2013; Zhao B. et al., 2020). We rationalized that these activators could increase the rate of cGAMP production without necessarily altering the amount of SUMO-mcGAS inside the cells. Using M9 minimal medium, we supplemented each of these cations separately and tested the yield of the cGAMP in the supernatants. None of the cation supplements increased the yield of cGAMP: supplementation of Zn^2+^ yielded 150 ± 20 mg/L, while Mn^2+^ surprisingly decreased the amount of cGAMP in the supernatant (50 ± 20 mg/L) without altering cell growth (Figure 3G; Supplementary Table S1).

**Figure 4:**
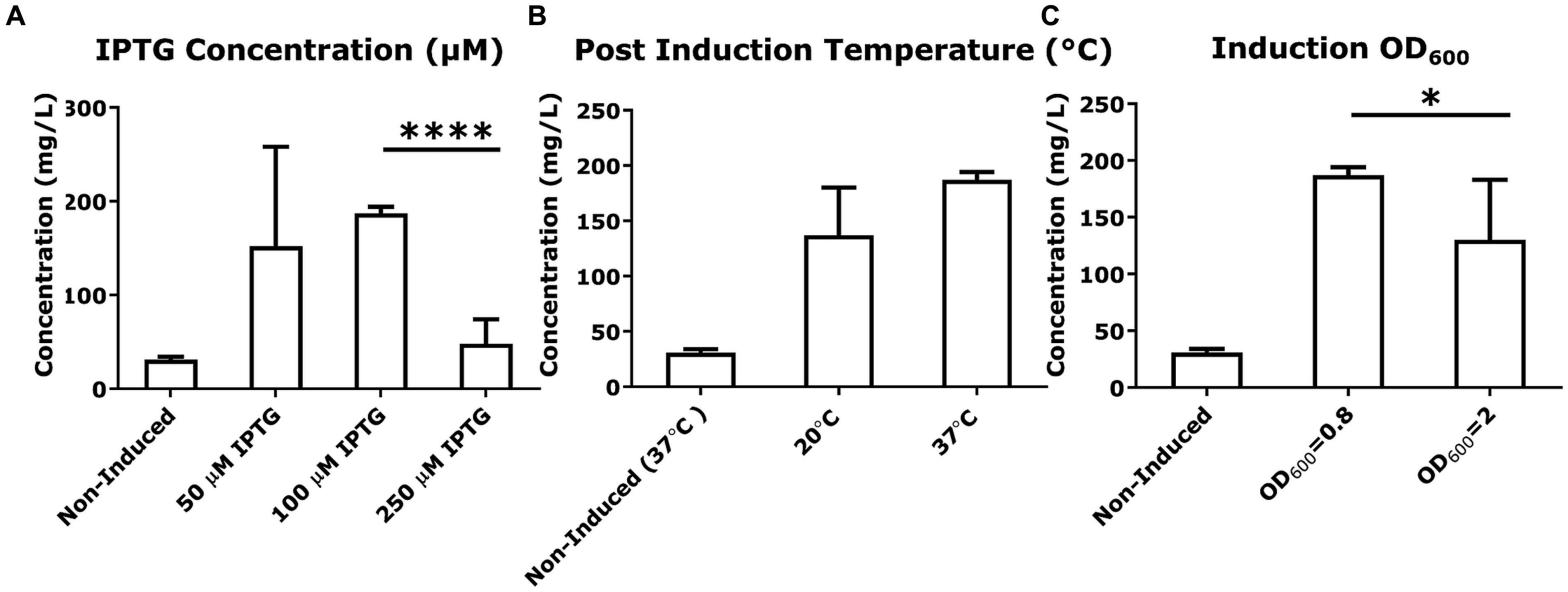
Variation in culture conditions to optimize cGAMP productivity. **(A–C)** The concentration of cGAMP in the *E. coli* BL21(DE3) culture supernatants was measured using HPLC at 256 nm. The comparison of cGAMP concentrations among variations in culture conditions included IPTG concentrations used for induction, post-induction culture temperature, and cell number [the optical density (OD_600_) of culture] at induction. All cultures were grown in M9 minimal medium. * Bar plots show the average measurement done over three independent cultures (*N* = 3) and the error bars show the SEM. Statistical significance indicated by (*) was performed for dataset comparisons using unpaired, two-tailed t-test and Welch’s correction.

To explore if altering the expression of SUMO-mcGAS would alter the production and secretion of cGAMP, we tested a range of inducer (IPTG) concentrations (50, 100, 250 μM). However, all of these conditions uniformly decreased the yield of secreted cGAMP compared to standard conditions using 100 μM IPTG, with 50 μM and 250 μM IPTG addition yielding 150 ± 100 mg/L and 50 ± 30 mg/L, respectively, (Figure 4A). To determine whether the DNA binding activity of mcGAS was affecting cell growth upon induction (enzyme expression did not impact cell viability), we also varied the optical density (OD_600_) at which the bacterial culture was induced. Late induction did not improve cGAMP yield (130 ± 50 mg/L at OD_600_ = 2 vs. 186 ± 7 mg/L at OD_600_ = 0.8; Figure 4B). Lowering the post-induction temperature of bacterial cultures producing recombinant proteins to improve productivity is a widely used practice (Escher et al., 1989; Gadgil et al., 2005; Rolf et al., 2019), and so we tested whether the expression at a lower temperature (20°C) would impact the amount of folded SUMO-mcGAS and hence the yield of mcGAS but again the yield was not significantly different (140 ± 40 mg/L) compared to standard expression at 37°C (187 ± 7 mg/L; Figure 4C).

In summary, these optimization experiments identified that the highest yield of cGAMP in the supernatant of SUMO-mcGAS was obtained by culturing *E. coli* BL21(DE3) cells in M9 minimal medium at 37°C without supplementation with additional divalent cations.

### 3.2 Comparing the ability of plasmid and genomic DNA to activate mcGAS

Measuring mcGAS activity and the role that DNA plays in activating cGAS has been the subject of studies trying to understand the mcGAS activity in the context of the STING pathway (Gao et al., 2013) or the cell cycle (Zhao B. et al., 2020; Herzner et al., 2021). To explore the exact DNA molecules (plasmid vs. genome) that serve to activate mcGAS in the cytoplasm of *E. coli*, we performed a series of *in vitro* experiments comparing the ability of these DNA molecules to activate purified mcGAS. We fixed the amounts of mcGAS, ATP, and GTP while varying the amount of dsDNA added to the enzymatic reaction. The production of cGAMP (0.7–1.4 × 10^9^ molecules/molecule of dsDNA/ h) was constant across a range of concentrations of plasmid DNA (1–10 nM). By comparison, genomic DNA was able to produce 8 ± 1 × 10^10^ molecules of cGAMP/molecule of dsDNA/h at a concentration of 320 mg/L (0.1 nM). It is to be noted that the concentrations of dsDNA tested here resemble the theoretically calculated dsDNA concentrations in *E. coli*.

**Figure 5:**
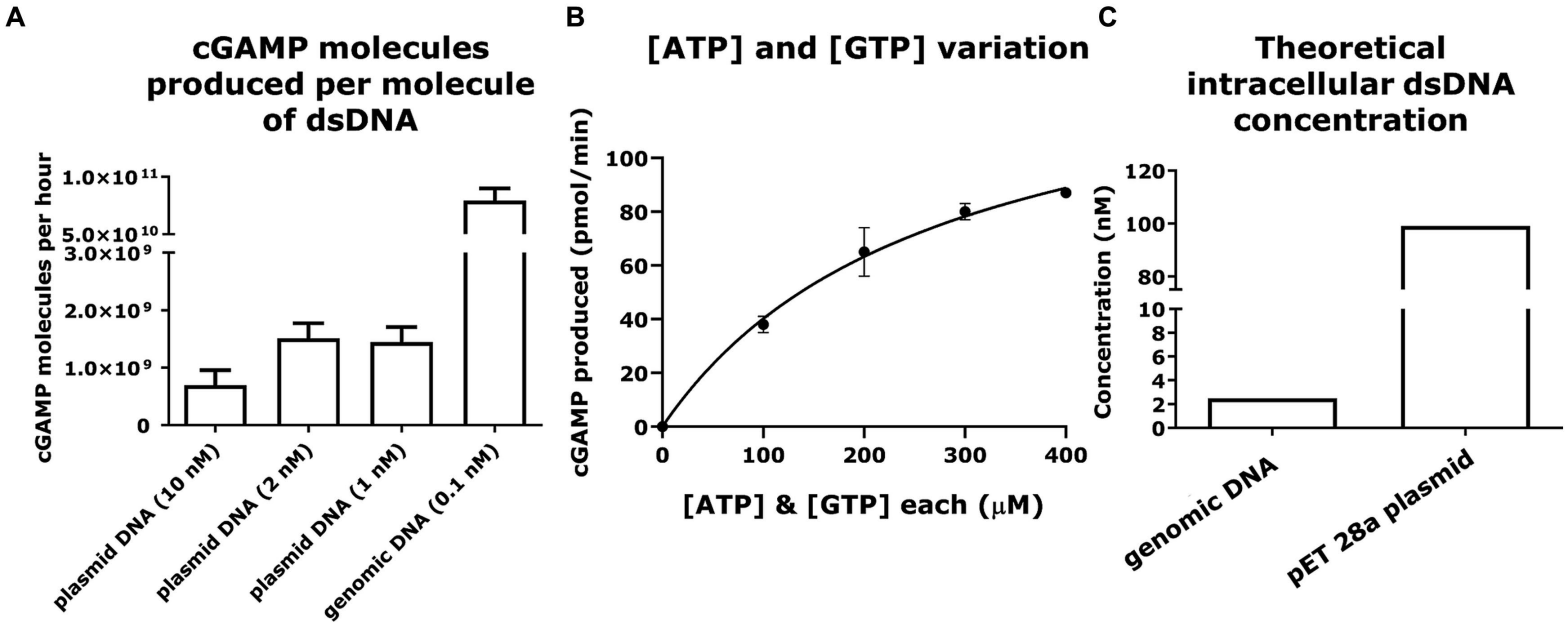
Plasmid and genomic DNA can elicit efficient cGAMP production via mcGAS at physiologically relevant concentrations. **(A)** The ability of dsDNA to efficiently activate mcGAS was quantified *in vitro*. The enzymatic reaction was carried out at fixed concentrations of ATP (100 μM), GTP (100 μM), and mcGAS (0.1 μM), and varying concentrations of dsDNA. The cGAMP produced was measured using the standard HPLC assay (256 nm). **(B)** The efficiency of various NTP (ATP/GTP) concentrations to activate mcGAS was quantified *in vitro.* The enzymatic reaction was carried out at varying concentrations of ATP (100, 200, 300, 400 μM), GTP (100, 200, 300, 400 μM), with fixed concentrations of mcGAS (0.1 μM) and dsDNA (2 nM). The cGAMP was measured using the standard HPLC assay (256 nm). **(C)** The theoretically calculated intracellular concentrations of double-stranded DNA plotted for plasmid DNA (pET 28a) and genomic DNA from *E. coli* BL21(DE3) (Elowitz et al., 1999; Wang et al., 2013). * Bar plots show the average measurement done over three replicates (*N* = 3) and the error bars show the SEM.

To contextualize these results, we calculated the molecules of cGAMP being produced using different amounts of dsDNA in each reaction mixture, keeping the amounts of mcGAS enzyme, ATP, and GTP constant. It was observed that per molecule of dsDNA; genomic DNA can produce higher quantities of cGAMP in reaction mixture compared to that produced by plasmid (Figure 5A). This is probably because genomic DNA harbors more mcGAS binding sites than plasmid DNA due to the considerable differences in the length of the dsDNA. When we compared the size of plasmid and genomic dsDNA, we found that cGAMP molecules binding per base pair of dsDNA was lower for genomic DNA compared to plasmid (2 ± 0.2 × 10^4^ vs. 17 ± 4 × 10^4^ molecules of cGAMP/base pair of dsDNA). The lower activation by the genomic DNA could be attributed to its highly condensed structure precluding uniform binding by mcGAS.

We also wanted to confirm that *in vivo* concentrations of ATP and GTP in *E. coli* would be optimum for mcGAS activation. Toward this, we conducted the enzymatic reaction at varying concentrations of ATP and GTP, with fixed concentrations of mcGAS and plasmid dsDNA. We observed that the enzymatic production of cGAMP followed standard Michaelis–Menten kinetics (Figure 5B). The estimated kinetic parameters were consistent with previous studies of mcGAS activity confirming that plasmid DNA mediated mcGAS activation does not compromise its catalytic efficiency (Vincent et al., 2017). Second, from these kinetic data, it is apparent that at the known intracellular concentrations of ATP (~3,500 μM) and GTP (~1700 μM) in *E. coli*, we anticipate saturation of mcGAS kinetics (Buckstein et al., 2008). We thus concluded that the intracellular substrate concentrations would not kinetically limit cGAMP productivity. We next evaluated if *in vivo* concentrations of the plasmid and genomic DNA would be sufficient to activate mcGAS effectively, and not cause a bottleneck in mcGAS activation and subsequent cGAMP production. Approximating the values of *E. coli* cell volume (Wang et al., 2013) and the amount of DNA in an *E. coli* cell (Elowitz et al., 1999), we concluded that the concentrations of both plasmid and genomic DNA we tested were well within the physiological range of those found in *E. coli* BL21 (DE3) cells (Figure 5C). As the amounts of dsDNA tested here resemble the theoretically calculated dsDNA concentrations in *E. coli*, we thus concluded that genomic DNA or plasmid DNA would not be a bottleneck to cGAMP production.

### 3.3 Impact of altering the DNA-binding of mcGAS on the yield of cGAMP in *Escherichia coli* culture supernatants

**Figure 6:**
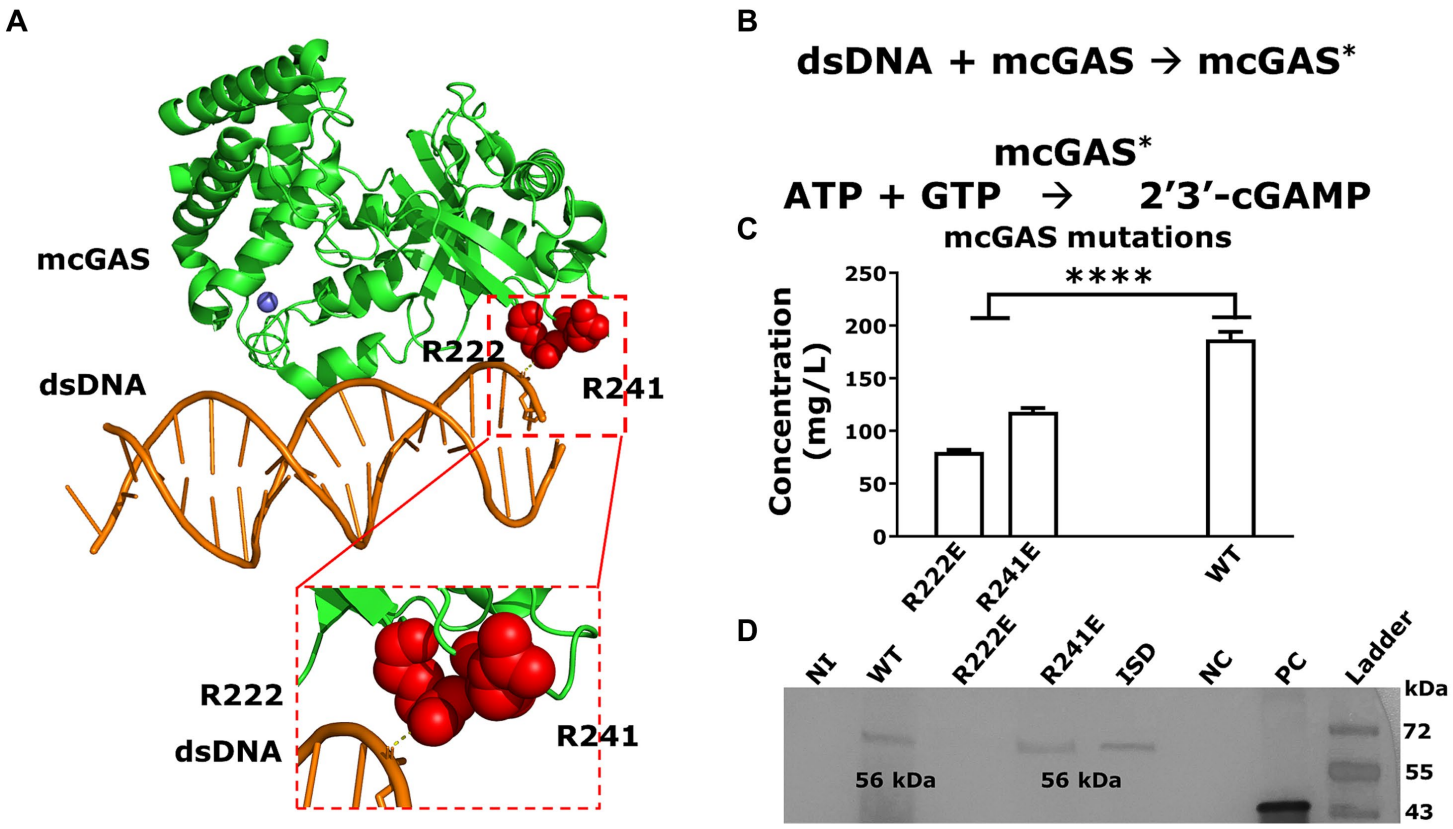
Abolishing DNA binding of mcGAS significantly decreased cGAMP yields. **(A)** The crystal structure of mcGAS (PDB: 406A; Zhang et al., 2014), rendered using PyMol (version 2.5.4). The enzyme is colored green and the two Arg (Arg222 and Arg241) within the DNA binding domain that make electrostatic interactions with the dsDNA (orange), are shown as spheres in red. **(B)** The two-step sequential reaction for the synthesis of cGAMP by mcGAS. **(C)** The concentration of cGAMP in the *E. coli* BL21(DE3) supernatants harboring the different mcGAS variants was measured using HPLC assay at 256 nm. **(D)** The expression of mcGAS and mcGAS variants was determined using Western blotting using an anti-SUMO antibody. The positive control was a SUMO-tagged protein with a molecular weight of 42 kDa. * Bar plots show the average measurement done over three replicates (*N* = 3) and the error bars show the SEM. Statistical significance indicated by (*) was performed for dataset comparisons using unpaired, two-tailed t-test and Welch’s correction.

mcGAS binds to dsDNA to change into the catalytically active conformation to synthesize cGAMP (Figures 6A,B). We next explored whether amino acid substitutions in mcGAS that are known to abolish mcGAS-dsDNA binding for mcGAS-catalyzed cGAMP synthesis (i.e., confer constitutive activity) could increase the yield of cGAMP in *E. coli* supernatants (Volkman et al., 2019). Accordingly, we cloned two mutants of mcGAS, encoding Arg222Glu (R222E) and Arg241Glu (R241E) variants (Figure 6A) into the pET28a-SUMO-mcGAS plasmid.

We transformed these variants into *E. coli* BL21(DE3) cells and produced cGAMP under the optimized culture conditions listed above. The growth of *E. coli* BL21(DE3) cells transformed with either the mcGAS-Arg222Glu or mcGAS-Arg241Glu variants was no different from cells transformed with wt-mcGAS (Supplementary Table S1). When we tested the culture supernatants using HPLC, both the mcGAS- Arg222Glu and mcGAS-Arg241Glu variants yielded lower cGAMP (58 ± 6 mg/L and 118 ± 3 mg/L respectively) compared to the wt-mcGAS (186 ± 7 mg/mL; Figure 6C). The yield with the mcGAS- Arg222Glu variant is consistent with prior studies that showed that while this mutation eliminates the need for DNA-based activation, it reduces cGAMP yield ~3-fold (Volkman et al., 2019). The data from the mcGAS-Arg241Glu variant however was surprising since prior studies showed that this mutation abolishes DNA binding and improves cGAMP yield by 3-fold (Volkman et al., 2019). To test whether the reduced cGAMP productivity is due to lower enzyme expression, we performed a western blot on the cell lysates of *E. coli* BL21(DE3) cell culture. Western blotting showed that the mcGAS-Arg241Glu variant was expressed at levels equivalent to wt-mcGAS, whereas the mcGAS-Arg222Glu variant was not detectable (Figure 6D). Collectively, these experiments showed that while the low productivity of mcGAS-Arg222Glu variant can be due to poor expression in *E. coli*, the mcGAS-Arg241Glu variant was expressed at levels comparable to wt-mcGAS and yet did not yield higher amounts of cGAMP in the supernatant.

In *E. coli* bacterial culture, the wt-mcGAS in modified M9 medium thus outperformed all other media, variations to the culture, as well as mutations in the DNA binding region of mcGAS in yielding the highest titer of cGAMP.

### 3.4 Single-step anion exchange to purify cGAMP and remove LPS from bacterial supernatants

**Figure 7:**
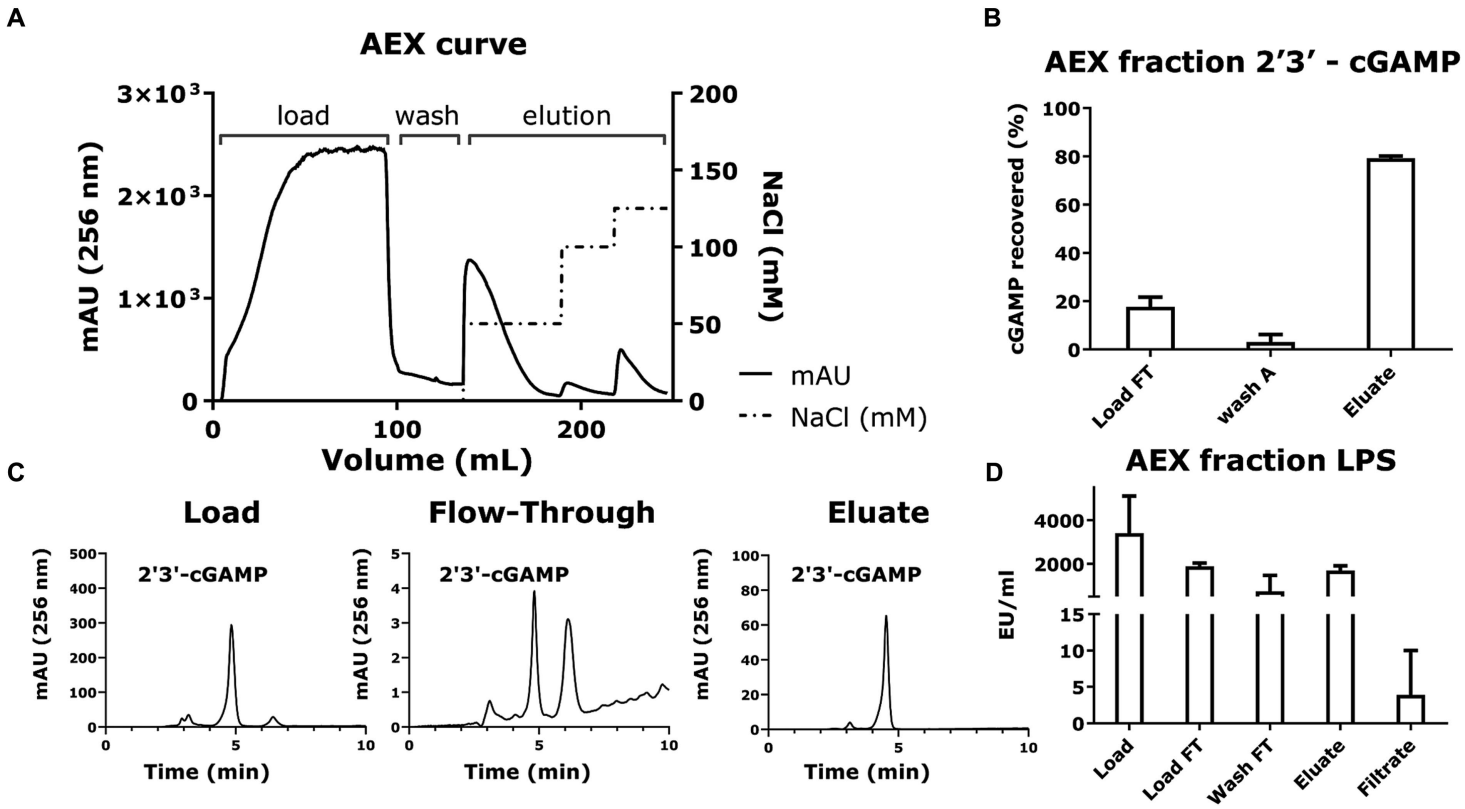
Anion exchange chromatography and LPS removal via filtration recovers 2′3’-cGAMP as the purified product. **(A)** The chromatogram plotting volume (mL) against Absorbance (mAU at 256 nm) and NaCl concentration (mM) shows the overall anion exchange chromatography process for purifying cGAMP. The cGAMP-containing supernatant from the microbial cell culture was diluted and loaded onto the anion exchange column, and finally eluted with increasing concentrations of NaCl. **(B)** The yield of the cGAMP in the different fractions of anion exchange chromatography was measured using HPLC (256 nm). **(C)** Representative HPLC chromatograms (256 nm) of cGAMP within the different fractions from the anion exchange chromatography. **(D)** Quantification of LPS in the AEX fractions was determined using the Pierce Endotoxin kit. * Bar plots show the average measurement done over three replicates (*N* = 3) and the error bars show the SEM. Statistical significance indicated by (*) was performed for dataset comparisons using unpaired, two-tailed *t*-test and Welch’s correction.

There have been multiple strategies employed to purify cyclic dinucleotides, including the use of affinity exchange resins (Lv et al., 2019), ion exchange chromatography (Holleufer and Hartmann, 2018), and reverse phase chromatography (Lv et al., 2019). Our approach to cGAMP purification was to avoid the use of specialized resins and explore the use of anion exchange chromatography with increasing salt concentrations for elution (Figure 7A; Holleufer and Hartmann, 2018).

To reduce the conductivity of the load and avoid cGAMP losses in subsequent load flow-through, we diluted the bacterial supernatants with loading buffer, loaded them onto the column, and eluted the cGAMP with increasing buffer conductivity. The results (Figure 7B) revealed that the cGAMP was captured efficiently by the HighQ resin and was recovered in the eluate fractions (Figure 7C). The resulting eluate recovered 70 ± 1% of the loaded cGAMP.

Since LPS is a pyrogen and immunostimulant, contamination of cGAMP with LPS will preclude its use in any *in vitro* bioactivity assays and for all *in vivo* applications. The filtration step using a 3 kDa MWCO filtration setup removed LPS, reducing it down from <1,500 EU/mL prefiltration to a post-filtration value of <20 EU/mL (<0.3 EU/μg; Figure 7D). At the same time, the process retained 70 ± 1% loaded cGAMP as confirmed by HPLC (Figure 7B).

The purification and LPS removal steps yield cGAMP with a high final purity while ensuring that the final LPS concentration < 20 EU/mL (Figure 7D).

### 3.5 Purified cGAMP shows biological activity *in vitro*

To confirm the biological activity of the *E. coli* derived, purified cGAMP, we used a commercially available THP-1 reporter cell line. This modified THP-1 cell line is designed to conditionally secrete luciferase downstream of an interferon-responsive factor (IRF) promoter. Upon stimulation by cGAMP, these THP-1 cells would produce luciferase in the conditioned supernatant, serving as an indicator of IRF activation (Supplementary Figure 2A). We observed that the luminescence induced by our final cGAMP product was significantly higher [34,000 ± 5,000 Relative Fluorescence Units (RLU)] than the non-induced mcGAS cell culture supernatant filtered through the 3 kDa MWCO filter (610 ± 10 RLU; Supplementary Figure 2B). Moreover, the measured luminescence in THP-1 cells with the final cGAMP product was comparable to the chemically synthesized commercial cGAMP, thus confirming that our product was biologically active and retained its ability to stimulate an IRF response in the THP-1 cell line. In summary, we were able to manufacture, purify, and confirm the biological activity of cGAMP using an *E. coli* based whole cell biocatalysis.

## 4 Discussion

We aimed to design a recombinant method based on the ability of mcGAS to produce and purify cGAMP in an *E. coli* expression system. The expression, regulation, and post-translational modifications (PTM) of mcGAS in mammalian cells have been studied extensively over the past decade (Rolf et al., 2019; Volkman et al., 2019; Li et al., 2021; Zhong and Shu, 2021; Song et al., 2022). In mammalian cells, the PTMs of mcGAS influence a variety of factors, including its ability to bind to dsDNA, its structure, and its stability. For instance, phosphorylation is one of the major contributors to mcGAS deactivation during mitosis. The Ser291 residue in mcGAS (Ser305 in human cGAS) when phosphorylated, inhibits the ability of mcGAS to bind to genomic DNA, and thus cannot induce the conformational changes necessary to recruit ATP and GTP at its substrate binding sites (Song et al., 2022). The vast majority of PTMs in mammalian cells are utilized to deactivate the mcGAS; by contrast, the activation of mcGAS is facilitated by the addition of the SUMO tag and allosteric binding by dsDNA (Dai et al., 2019; Wu and Li, 2020). It is for this reason that we decided to clone the mcGAS as a SUMO fusion and attempt expression within the cytoplasm of *E. coli* wherein the enzyme has direct access to dsDNA (plasmid and/or genome). We posited, and our data confirmed, that mcGAS can be activated efficiently by *E. coli* derived genomic and/or plasmid DNA.

To the best of our knowledge, a study comparing the relative efficiencies of genomic and plasmid DNA to activate mcGAS *in vitro* has not been reported. Previous studies have calculated the K_M_ values of ATP and GTP concerning mcGAS (Vincent et al., 2017), as well as studies on mcGAS activities at various concentrations of dsDNA, usually a 45-base pair immunostimulatory sequence (Zhou et al., 2018). Our results demonstrate that at least at physiologically relevant concentrations both genomic and plasmid DNA are efficient activators of mcGAS leading to efficient production of cGAMP in *E. coli* without the need for addition of exogenous DNA. Our cellular manufacturing platform benefits from three features. First, the crowded environment of *E. coli* cytosol likely facilitates reaction kinetics of our multi-component reaction by enabling the close proximity of the mcGAS enzyme, the plasmid/genomic DNA activator, and the substrates (ATP/GTP) (Ladkau et al., 2014). Second, the intracellular concentrations of both substrates, ATP and GTP, are higher than the K_M_ of mcGAS for each of these substrates, and this permits saturation kinetics within the cells. Third, the product, cGAMP, is secreted into the supernatant, and this facilitates driving the reaction forward because of the intracellular depletion of the product.

To optimize the production of cGAMP, we introduced mutations within the DNA binding domain of mcGAS to render it constitutively active and abolish DNA dependence. We surprisingly observed that both the Arg241Glu mcGAS variant and Arg222Glu mcGAS variant showed overall low productivity in the whole cell cGAMP production process. While the relatively low expression of mcGAS Arg222Glu variant in *E. coli* cells might be responsible for the low cGAMP productivity, further studies are needed to establish the exact reasons for the low productivity of the Arg241Glu mcGAS variant. We hypothesize that since wt-mcGAS has been shown to phase separate on dsDNA inside cells (Du and Chen, 2018; Xiao et al., 2022), mutations that prevent binding to dsDNA can also prevent phase separation and hence decrease the overall enzymatic productivity. In the broader picture, the number of DNA sites or DNA binding is not limiting for the production of cGAMP by mcGAS in *E. coli* cells and introducing mutations to abolish DNA binding might not thus be beneficial to the yield of cGAMP.

There are other parameters available to optimize the yield of the secreted cGAMP that we have not tested. We have not mapped out the mechanism of export of cGAMP and whether the rate of export of cGAMP from *E. coli* can be improved by overexpression of one or more transporters. As with other heterologous metabolic pathways, it is unlikely that we have reached the optimal yield of cGAMP within the cell or within the supernatants (Ma et al., 2011). Systematic screening for *E. coli* phosphodiesterases that can potentially hydrolyze cGAMP can identify genome knockout strategies that can improve the yield of cGAMP.

Downstream processing of recombinant proteins and peptides is often a complex process including multiple purification and polishing steps leading to a large environmental footprint (Liu et al., 2010). With these factors in mind, we designed a downstream process that is simple, efficient, and cost-effective. The simplicity derives from the use of a conventional bacterial culture, followed by a single-step purification. Strategies like eschewing the use of expensive proprietary strains like *E. coli* BL21 (DE3) CodonPlus RIL cells and expensive affinity-based columns helped us make the process comparatively economical. In the latter case, we used a commercially available anion exchange resin instead of STING-affinity-based columns (Lv et al., 2019), the manufacturing of which would be an added expense to the purification process. Moreover, the binding capacity of HighQ resin would be higher than that of STING-Ligand Binding Domain (LBD), which has a 1:1 stoichiometric binding ratio to cGAMP, making the overall resin requirement higher, thus driving the cost further up. Finally, by incorporating an LPS removal step (Sandle, 2016), we devised a production process for cGAMP that contains minimal LPS and can be used as part of formulations for activating immune cells *in vitro* and *in vivo* (Leekha et al., 2022).

The analytical methods that we have used for routine characterization based on HPLC and LC–MS are well suited for large scale synthesis. For routine use in small scale and research settings, alternative equipment-free methods based for the detection of cGAMP based on ELISA can be implemented. At the research scale, we (one graduate student) are able to produce 2 mg of cGAMP from a standard 20 mL culture within 1 day of harvesting the cells and the economic cost of this research-grade process is favorable compared to the market price of 1 mg of chemically synthesized, LPS free cGAMP (~$500).

In summary, our study focuses on the single-step purification process for cGAMP using anion exchange chromatography, minimizing LPS, and increasing cGAMP yield. Our results show that the microbial-based preparation method of cGAMP is relevant due to the ease of cultivating bacteria; and the simplicity of our purification procedure appeals to both large and small-scale processes. We believe that the method we described in this paper can be further developed to produce 2′3’-cGAMP on both large-scale manufacturing and small-scale research purposes.

## Data availability statement

The raw data supporting the conclusions of this article will be made available by the authors, without undue reservation.

## Ethics statement

Ethical approval was not required for the studies on humans in accordance with the local legislation and institutional requirements because only commercially available established cell lines were used. Ethical approval was not required for the studies on animals in accordance with the local legislation and institutional requirements because only commercially available established cell lines were used.

## Author contributions

RK: Conceptualization, Writing – original draft, Writing – review & editing, Data curation, Formal analysis, Investigation, Methodology, Software, Validation. VM: Data curation, Methodology, Software, Writing – review & editing. NN: Data curation, Methodology, Software, Writing – review & editing. PC: Resources, Supervision, Writing – review & editing. RW: Resources, Supervision, Writing – review & editing. NV: Conceptualization, Funding acquisition, Project administration, Software, Writing – original draft, Writing – review & editing.

## References

[ref1] An X. Martinez-Paniagua M. Rezvan A. Sefat S. R. Fathi M. Singh S. . (2021). Single-dose intranasal vaccination elicits systemic and mucosal immunity against SARS-CoV-2. iScience 24:103037. doi: 10.1016/j.isci.2021.103037, PMID: 34462731 PMC8388188

[ref2] Barber G. N. (2015). STING: infection, inflammation and cancer. Nat. Rev. Immunol. 15, 760–770. doi: 10.1038/nri3921, PMID: 26603901 PMC5004891

[ref3] Bartsch T. Becker M. Rolf J. Rosenthal K. Lutz S. (2022). Biotechnological production of cyclic dinucleotides-challenges and opportunities. Biotechnol. Bioeng. 119, 677–684. doi: 10.1002/bit.28027, PMID: 34953086

[ref4] Becker M. Lutz S. Rosenthal K. (2021a). Environmental assessment of enzyme production and purification. Molecules 26:573. doi: 10.3390/molecules26030573, PMID: 33499126 PMC7865607

[ref5] Becker M. Nikel P. Andexer J. N. Lutz S. Rosenthal K. (2021b). A multi-enzyme Cascade reaction for the production of 2′3'-cGAMP. Biomol. Ther. 11:590. doi: 10.3390/biom11040590, PMID: 33923845 PMC8073963

[ref6] Becker M. Ziemińska-Stolarska A. Markowska D. Lütz S. Rosenthal K. (2023). Comparative life cycle assessment of chemical and biocatalytic 2′3’-cyclic GMP-AMP synthesis. Chem Sus Chem 16:e202201629. doi: 10.1002/cssc.202201629, PMID: 36416867

[ref7] Buckstein M. H. He J. Rubin H. (2008). Characterization of nucleotide pools as a function of physiological state in *Escherichia coli*. J. Bacteriol. 190, 718–726. doi: 10.1128/JB.01020-07, PMID: 17965154 PMC2223692

[ref8] Carozza J. A. Bohnert V. Nguyen K. C. Skariah G. Shaw K. E. Brown J. A. . (2020). Extracellular cGAMP is a cancer cell-produced immunotransmitter involved in radiation-induced anti-cancer immunity. Nat Cancer 1, 184–196. doi: 10.1038/s43018-020-0028-4, PMID: 33768207 PMC7990037

[ref9] Chen C. Xu P. (2022). Cellular functions of cGAS-STING signaling. Trends Cell Biol. 33, 630–648. doi: 10.1016/j.tcb.2022.11.001 36437149

[ref10] Dai J. Huang Y. J. He X. Zhao M. Wang X. Liu Z. S. . (2019). Acetylation blocks cGAS activity and inhibits self-DNA-induced autoimmunity. Cell 176, 1447–1460.e14. doi: 10.1016/j.cell.2019.01.016, PMID: 30799039 PMC8274936

[ref11] Du M. Chen Z. J. (2018). DNA-induced liquid phase condensation of cGAS activates innate immune signaling. Science 361, 704–709. doi: 10.1126/science.aat1022, PMID: 29976794 PMC9417938

[ref12] Elowitz M. B. Surette M. G. Wolf P. E. Stock J. B. Leibler S. (1999). Protein mobility in the cytoplasm of *Escherichia coli*. J. Bacteriol. 181, 197–203. doi: 10.1128/JB.181.1.197-203.1999, PMID: 9864330 PMC103549

[ref13] Escher A. O'Kane D. J. Lee J. Szalay A. A. (1989). Bacterial luciferase alpha beta fusion protein is fully active as a monomer and highly sensitive in vivo to elevated temperature. Proc. Natl. Acad. Sci. 86, 6528–6532. doi: 10.1073/pnas.86.17.6528, PMID: 2671993 PMC297877

[ref14] Gadgil M. Kapur V. Hu W.-S. (2005). Transcriptional response of *Escherichia coli* to temperature shift. Biotechnol. Prog. 21, 689–699. doi: 10.1021/bp049630l, PMID: 15932244

[ref15] Gaffney B. L. Veliath E. Zhao J. Jones R. A. (2010). One-flask syntheses of c-di-GMP and the [Rp,Rp] and [Rp,Sp] thiophosphate analogues. Org. Lett. 12, 3269–3271. doi: 10.1021/ol101236b, PMID: 20572672 PMC2905038

[ref16] Gao P. Ascano M. Wu Y. Barchet W. Gaffney B. L. Zillinger T. . (2013). Cyclic [G(2′,5′)pA (3′,5′)p] is the metazoan second messenger produced by DNA-activated cyclic GMP-AMP synthase. Cell 153, 1094–1107. doi: 10.1016/j.cell.2013.04.046, PMID: 23647843 PMC4382009

[ref17] Gogoi H. Mansouri S. Jin L. (2020). The age of cyclic dinucleotide vaccine adjuvants. Vaccines (Basel) 8:453. doi: 10.3390/vaccines8030453 32823563 PMC7563944

[ref18] Herzner A.-M. Schlee M. Bartok E. (2021). The many faces of cGAS: how cGAS activation is controlled in the cytosol, the nucleus, and during mitosis. Signal Transduct. Target. Ther. 6:260. doi: 10.1038/s41392-021-00684-3, PMID: 34244470 PMC8270980

[ref19] Holleufer A. Hartmann R. (2018). A highly sensitive anion exchange chromatography method for measuring cGAS activity in vitro. Bioprotocol 8:e3055. doi: 10.21769/BioProtoc.3055, PMID: 34532524 PMC8342075

[ref20] Hooy R. M. Massaccesi G. Rousseau K. E. Chattergoon M. A. Sohn J. (2020). Allosteric coupling between Mn2+ and dsDNA controls the catalytic efficiency and fidelity of cGAS. Nucleic Acids Res. 48, 4435–4447. doi: 10.1093/nar/gkaa084, PMID: 32170294 PMC7192592

[ref21] Hopfner K. P. Hornung V. (2020). Molecular mechanisms and cellular functions of cGAS-STING signalling. Nat. Rev. Mol. Cell Biol. 21, 501–521. doi: 10.1038/s41580-020-0244-x 32424334

[ref22] Hu M. M. Yang Q. Xie X. Q. Liao C. Y. Lin H. Liu T. T. . (2016). Sumoylation promotes the stability of the DNA sensor cGAS and the adaptor STING to regulate the kinetics of response to DNA virus. Immunity 45, 555–569. doi: 10.1016/j.immuni.2016.08.014, PMID: 27637147

[ref23] Ladkau N. Schmid A. Bühler B. (2014). The microbial cell-functional unit for energy dependent multistep biocatalysis. Curr. Opin. Biotechnol. 30, 178–189. doi: 10.1016/j.copbio.2014.06.003, PMID: 25035941

[ref24] Leekha A. Saeedi A. Kumar M. Sefat S. R. Martinez-Paniagua M. Fathi M. . (2022). An intranasal nanoparticle STING agonist has broad protective immunity against respiratory viruses and variants. bioRxiv 2022:488695. doi: 10.1101/2022.04.18.488695

[ref25] Li T. Huang T. Du M. Chen X. Du F. Ren J. . (2021). Phosphorylation and chromatin tethering prevent cGAS activation during mitosis. Science 371:eabc5386. doi: 10.1126/science.abc5386 33542149 PMC8171060

[ref26] Liu H. F. Ma J. Winter C. Bayer R. (2010). Recovery and purification process development for monoclonal antibody production. MAbs 2, 480–499. doi: 10.4161/mabs.2.5.12645, PMID: 20647768 PMC2958570

[ref27] Lv Y. Sun Q. Wang X. Lu Y. Li Y. Yuan H. . (2019). Highly efficient preparation of cyclic dinucleotides via engineering of dinucleotide Cyclases in *Escherichia coli*. Front. Microbiol. 10:2111. doi: 10.3389/fmicb.2019.02111, PMID: 31572324 PMC6753226

[ref28] Ma S. M. Garcia D. E. Redding-Johanson A. M. Friedland G. D. Chan R. Batth T. S. . (2011). Optimization of a heterologous mevalonate pathway through the use of variant HMG-CoA reductases. Metab. Eng. 13, 588–597. doi: 10.1016/j.ymben.2011.07.001, PMID: 21810477

[ref29] Petrovic M. Borchard G. Jordan O. (2021). Considerations for the delivery of STING ligands in cancer immunotherapy. J. Control. Release 339, 235–247. doi: 10.1016/j.jconrel.2021.09.033, PMID: 34592386

[ref30] Ritchie C. Carozza J. A. Li L. (2022). Biochemistry, cell biology, and pathophysiology of the innate immune cGAS-cGAMP-STING pathway. Annu. Rev. Biochem. 91, 599–628. doi: 10.1146/annurev-biochem-040320-101629, PMID: 35287475

[ref31] Ritchie C. Cordova A. F. Hess G. T. Bassik M. C. Li L. (2019). SLC19A1 is an importer of the Immunotransmitter cGAMP. Mol. Cell 75, 372–381.e5. doi: 10.1016/j.molcel.2019.05.006, PMID: 31126740 PMC6711396

[ref32] Rolf J. Siedentop R. Lutz S. Rosenthal K. (2019). Screening and identification of novel cGAS homologues using a combination of in vitro and in vivo protein synthesis. Int. J. Mol. Sci. 21:105. doi: 10.3390/ijms21010105, PMID: 31877895 PMC6981698

[ref33] Rosenthal K. Becker M. Rolf J. Siedentop R. Hillen M. Nett M. . (2020). Catalytic promiscuity of cGAS: a facile enzymatic synthesis of 2′-3'-linked cyclic dinucleotides. Chembiochem 21, 3225–3228. doi: 10.1002/cbic.202000433, PMID: 32633874 PMC7754487

[ref34] Sandle T. (2016). “11- endotoxin and pyrogen testing” in Pharmaceutical Microbiology. ed. Sandle T. (Oxford: Woodhead Publishing), 131–145.

[ref35] Song J. X. Villagomes D. Zhao H. Zhu M. (2022). cGAS in nucleus: the link between immune response and DNA damage repair. Front. Immunol. 13:1076784. doi: 10.3389/fimmu.2022.1076784, PMID: 36591232 PMC9797516

[ref36] Vincent J. Adura C. Gao P. Luz A. Lama L. Asano Y. . (2017). Small molecule inhibition of cGAS reduces interferon expression in primary macrophages from autoimmune mice. Nat. Commun. 8:750. doi: 10.1038/s41467-017-00833-9, PMID: 28963528 PMC5622107

[ref37] Volkman H. E. Cambier S. Gray E. E. Stetson D. B. (2019). Tight nuclear tethering of cGAS is essential for preventing autoreactivity. eLife 8:491. doi: 10.7554/eLife.47491, PMID: 31808743 PMC6927687

[ref38] Wang C. Guan Y. Lv M. Zhang R. Guo Z. Wei X. . (2018). Manganese increases the sensitivity of the cGAS-STING pathway for double-stranded DNA and is required for the host defense against DNA viruses. Immunity 48, 675–687.e7. doi: 10.1016/j.immuni.2018.03.017, PMID: 29653696

[ref39] Wang L. Zhou Y. J. Ji D. Zhao Z. K. (2013). An accurate method for estimation of the intracellular aqueous volume of *Escherichia coli* cells. J. Microbiol. Methods 93, 73–76. doi: 10.1016/j.mimet.2013.02.006, PMID: 23481146

[ref40] Wu Y. T. Fang Y. Wei Q. Shi H. Tan H. Deng Y. . (2022). Tumor-targeted delivery of a STING agonist improvescancer immunotherapy. Proc. Natl. Acad. Sci. USA 119:e2214278119. doi: 10.1073/pnas.2214278119, PMID: 36442099 PMC9894229

[ref41] Wu Y. Li S. (2020). Role of post-translational modifications of cGAS in innate immunity. Int. J. Mol. Sci. 21:7842. doi: 10.3390/ijms21217842, PMID: 33105828 PMC7660100

[ref42] Xiao Q. McAtee C. K. Su X. (2022). Phase separation in immune signalling. Nat. Rev. Immunol. 22, 188–199. doi: 10.1038/s41577-021-00572-5, PMID: 34230650 PMC9674404

[ref43] Zhang X. Wu J. Du F. Xu H. Sun L. Chen Z. . (2014). The cytosolic DNA sensor cGAS forms an oligomeric complex with DNA and undergoes switch-like conformational changes in the activation loop. Cell Rep. 6, 421–430. doi: 10.1016/j.celrep.2014.01.003, PMID: 24462292 PMC3969844

[ref44] Zhao Z. Ma Z. Wang B. Guan Y. Su X. D. Jiang Z. (2020). Mn (2+) directly activates cGAS and structural analysis suggests Mn(2+) induces a noncanonical catalytic synthesis of 2′3'-cGAMP. Cell Rep. 32:108053. doi: 10.1016/j.celrep.2020.108053, PMID: 32814054

[ref45] Zhao B. Xu P. Rowlett C. M. Jing T. Shinde O. Lei Y. . (2020). The molecular basis of tight nuclear tethering and inactivation of cGAS. Nature 587, 673–677. doi: 10.1038/s41586-020-2749-z, PMID: 32911481 PMC7704945

[ref46] Zhong L. Shu H.-B. (2021). Mitotic inactivation of the cGAS–MITA/STING pathways. J. Mol. Cell Biol. 13, 721–727. doi: 10.1093/jmcb/mjab061, PMID: 34609492 PMC8718187

[ref47] Zhou W. Whiteley A. T. de Oliveira Mann C. C. Morehouse B. R. Nowak R. P. Fischer E. S. . (2018). Structure of the human cGAS-DNA complex reveals enhanced control of immune surveillance. Cell 174, 300–311.e11. doi: 10.1016/j.cell.2018.06.026, PMID: 30007416 PMC6084792

