# Supplementary material for: The efficient synthesis and purification of 2′3’- cGAMP from Escherichia coli
Source: Front Microbiol. 2024 Mar 8;15:1345617. doi: 10.3389/fmicb.2024.1345617 (PMC10957790; doi:10.3389/fmicb.2024.1345617)
Supplement: Supplementary file 1 [file Data_Sheet_1.docx]

**Supplementary Information.**

**The efficient synthesis and purification of 2'3' cGAMP from *Escherichia coli***

**Rohan Kulkarni^1^, Vijay Maranholkar^1^, Nam Nguyen^1^, Patrick Cirino^1^, Richard C Willson^1^, Navin Varadarajan^1*^**


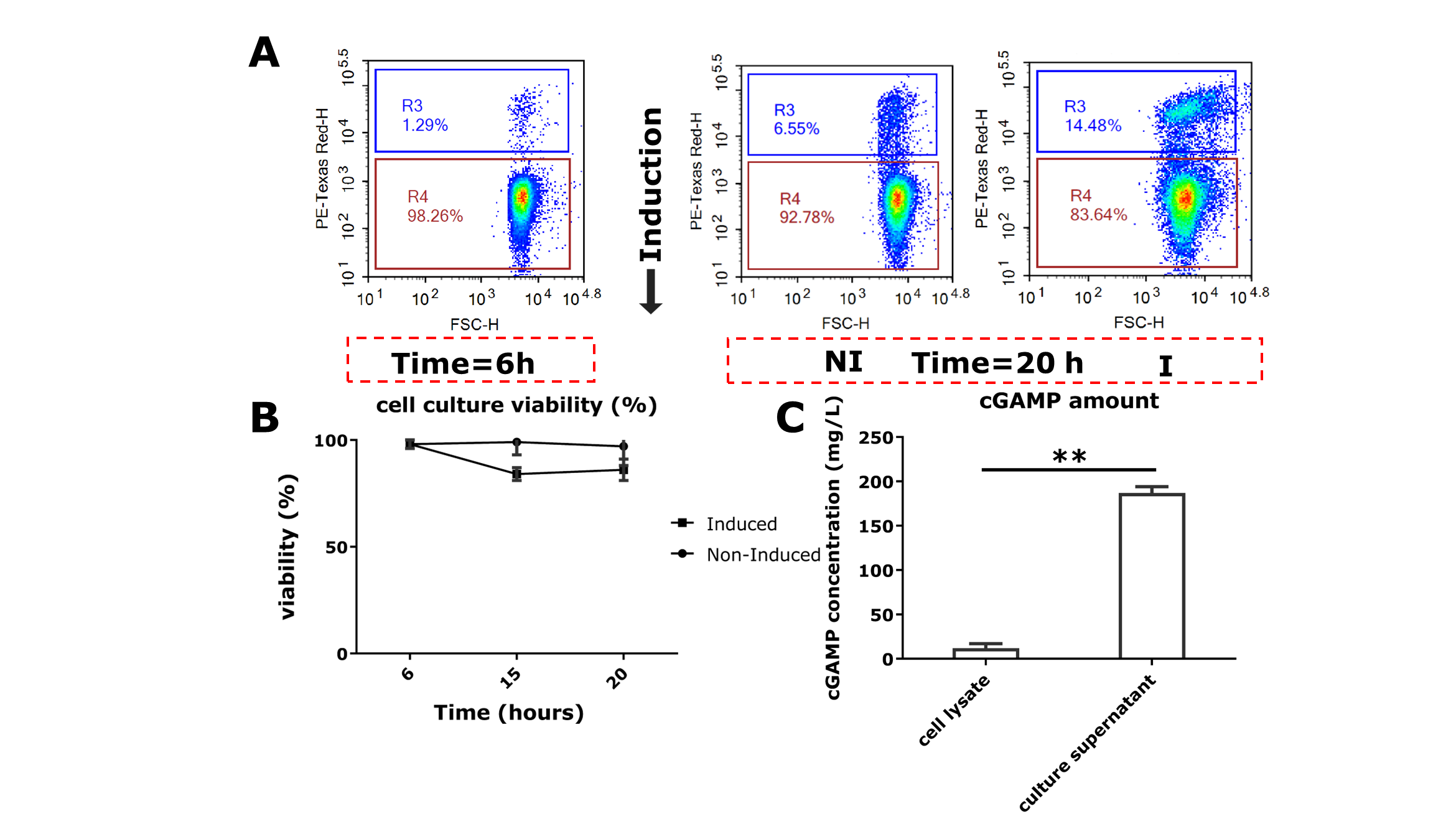
**Supplementary Figure 1: cGAMP productivity is not dependent on cGAMP released through cell death.**

(A) The flow cytometry experiments were done with a NovoCyte Flow Cytometer, NovoCyte 3000RYB (ACEA Biosciences Inc., San Diego, CA). The flow cytometry data from PI staining for bacterial cell culture is used to quantify viability of bacterial cells in samples as indicated in the dot plots. As PI is a viability indicating dye exhibiting fluorescence in the PE Texas Red region, higher PE Texas Red levels (>5 x 10^3^ RLU) indicate cell death. Samples (left to right) are WT-mcGAS in M9 medium at t=6 hours (before induction) and non-induced and induced WT-mcGAS in M9 medium at t=20 hours.

(B) Cell culture viability is shown for non-induced and induced cell culture for wt-mcGAS in M9 medium at different time points by quantifying the fractions of live cells from the flow cytometry assay.

(C) The cGAMP concentration was quantified in the intracellular (cell lysates) and extracellular (cell culture supernatant) fractions. Both fractions were derived from *E. coli* BL21(DE3) cells expressing wt-mcGAS cultured in M9 medium

* Bar and dot plots show the average measurement done over three replicates (N=3) and the error bars show the SEM. Statistical significance indicated by (*) was performed for dataset comparisons using unpaired, two-tailed t-test and Welch’s correction.


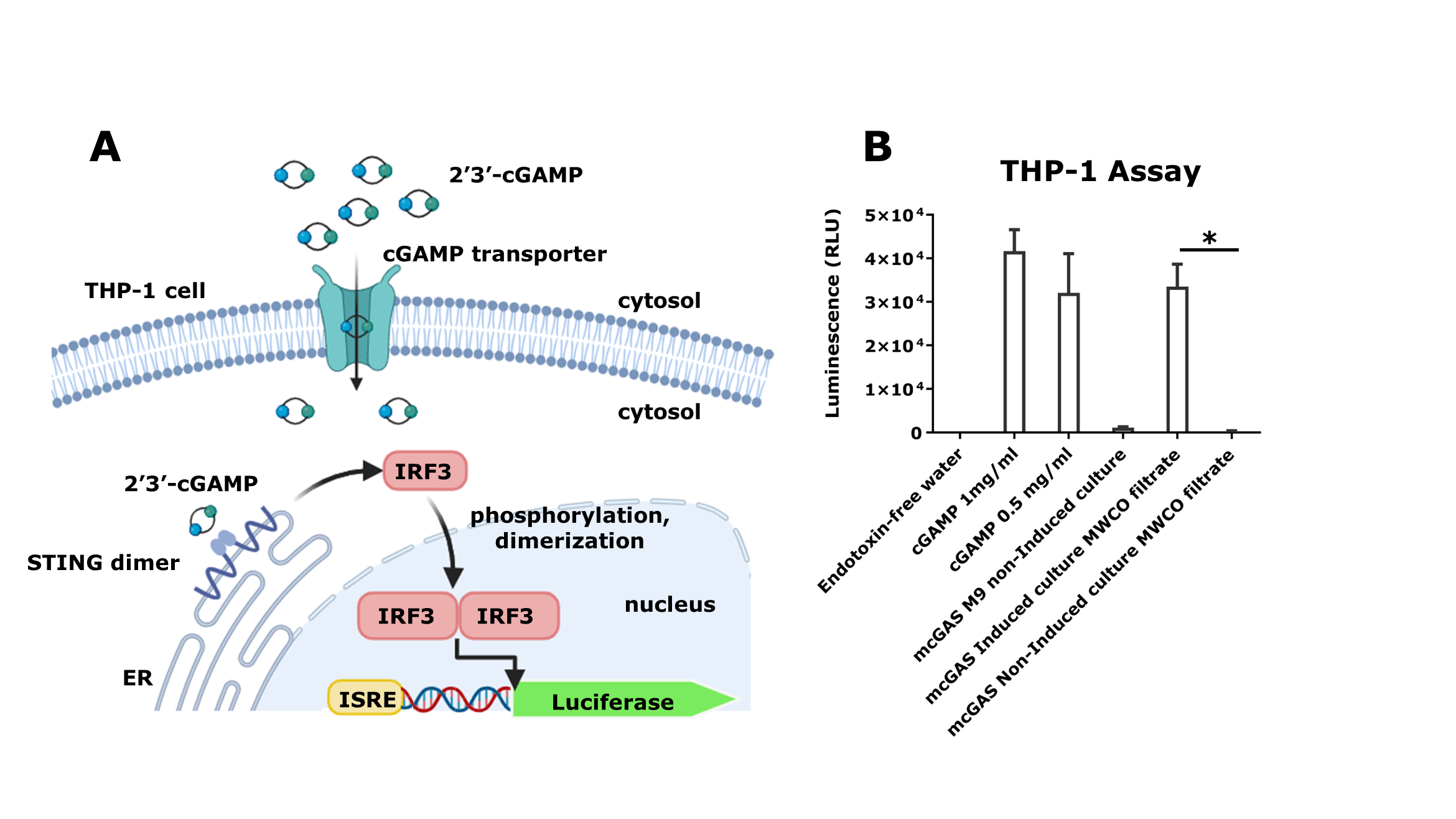
**Supplementary Figure 2:** **Purified cGAMP shows biological activity *in vitro.***

(A) Schematic illustrating the ability of cGAMP to activate the luciferase reporter in THP1 dual cells.

(B) The samples including endotoxin-free water (NC), Positive controls (chemically synthesized commercial cGAMP), the mcGAS cell culture supernatant and the 3 kDa MWCO-filtered final product were all tested for their ability to induce the THP-1 cell line to produce luciferase due to cGAMP-based activation. The measured luminescence (RLU) is compared amongst the samples.

* Bar plots show the average measurement done over three replicates (N=3) and the error bars show the SEM. Statistical significance indicated by (*) was performed for dataset comparisons using unpaired, two-tailed t-test and Welch’s correction.
